# From theory to practice: challenges and rewards of implementing ketogenic metabolic therapy in mental health

**DOI:** 10.3389/fnut.2024.1331181

**Published:** 2024-02-08

**Authors:** Nicole Laurent

**Affiliations:** Mental Health Keto, Vancouver, WA, United States

**Keywords:** ketogenic diet, metabolic psychiatry, KMT, ketogenic metabolic therapy, metabolic psychology, clinical psychology

## Abstract

This perspective article delves into the implementation of Ketogenic Metabolic Therapy (KMT) by a mental health counselor who attempts to bridge the gap between emerging research and real-world clinical application. Grounded in the author’s clinical experiences, the article communicates the potential of KMT in mental health care, highlighting both its therapeutic promise and the insights gained from hands-on patient interactions. While the adoption of KMT necessitates adjustments in societal, emotional, and dietary domains, especially within diverse mental health contexts, these challenges are surmountable with appropriate guidance and support. The article encourages the capture of qualitative data alongside quantitative measures and advocates for an approach that considers the broader implications of improved mental well-being on families and communities. As the field advances, interdisciplinary collaborations between researchers and clinicians will be pivotal in refining and expanding the application of KMT, ultimately enhancing patient outcomes and elevating the standard of mental health care.

## Introduction

While much of the existing literature on Ketogenic Metabolic Therapy (KMT) for mental health is grounded in emerging research (1–4), the perspective of a clinician, especially one with a background in clinical psychology, offers a distinctive viewpoint. Clinicians bridge the gap between theoretical research and its real-world application, providing valuable insights from their direct interactions with patients.

The true measure of a therapeutic approach is not just its empirical evidence but how it is applied and received in everyday clinical practice. This is especially true for mental health, where individual variability is vast. Hence, a practical understanding of KMT’s application, its challenges, and its real-world benefits is essential.

This leads us to the central question: “While the benefits of Ketogenic Metabolic Therapy (KMT) for mental health align well with research findings in a clinical setting, what are the practical barriers faced by patients and clinicians when attempting to implement KMT outside of research studies?”

In this perspective article, I aim to address this question primarily based on my clinical experiences. My goal is to highlight the opportunities and challenges of utilizing KMT for mental health, with a specific focus on a mental health clinician’s perspective.

## The therapeutic potential

In my clinical practice, I have been privileged to witness numerous success stories and remarkable transformations associated with Ketogenic Metabolic Therapy (KMT). These stories offer a glimpse into the immense therapeutic potential of KMT in the realm of mental health care.

One of these remarkable stories recently involved a 17-year-old who had been diagnosed with Schizoaffective disorder, suffered from both auditory and visual hallucinations, and was hospitalized for suicidal ideation. When I asked her what she wanted me to tell others about this diet, less than 8 weeks into her KMT treatment with me, she stated, “Just that this diet has been a miracle and a life changer for me.” Her firsthand account emphasizes the profound impact that KMT can have on individuals grappling with complex mental health conditions. It serves as a powerful testament to the hope and promise that KMT offers, particularly in the context of challenging disorders like Schizoaffective disorder.

However, this perspective article is not about case studies. Stories such as this often lead to questions about for whom and what mental health conditions might benefit from a ketogenic intervention. This patient responded very quickly and received great and sustained benefits. But there are many confounding variables in patient populations that I have worked with that make answering that question very difficult. Still, drawing from my clinical experience, I have gained insights into the nuanced challenges and considerations that arise when implementing KMT across various patient populations.

One aspect is the interaction between KMT and existing medications or substance use that most mental health populations are on when they come to seek treatment, which might pose unique challenges for patients. For example, inadequate management of potentiation effects or titration of medications can sometimes lead to unexpected difficulties. These experiences, observed in real-world clinical settings, underscore the need for prescriber training tailored to the nuances of KMT (5, 6). As KMT gains recognition as a treatment for mental health conditions, the importance of updated and appropriate training for prescribers becomes evident. The complexities of medication management and patient expectations necessitate a nuanced approach, highlighting the importance of bridging the gap between research and practice. Prescribers untrained in ketogenic metabolic therapy (KMT) might misinterpret potentiation effects or medication withdrawal symptoms as a relapse of the patient’s condition (6). For instance, a patient’s re-emergence of previously resolved symptoms could lead to unnecessary medication increases. Additionally, withdrawal effects from discontinued medications might be wrongly seen as a deterioration in mental health or KMT’s ineffectiveness (7–10).

It is crucial to emphasize that the field of KMT for mental health care is still evolving, and attempting to profile and determine who should or should not have access to KMT based on incomplete research findings or naïve clinical practices in the real world can be premature and potentially detrimental. Until clinical practice among prescribers working with this population matures, it becomes clear that, particularly in the context of psychiatric medications, a clear understanding of who does and does not benefit may not yet be possible (11). This will be a continued area of research as scientists attempt to discern what phenotypes and presentations of mental illness benefit most.

## Real-world challenges

### Transition challenges and emotional responses

The process of transitioning to Ketogenic Metabolic Therapy (KMT) involves significant changes in dietary habits, presenting patients with both practical and psychological challenges. Drawing from clinical experience, it’s essential to consider several key aspects of this transition. Patients often grapple with the initial difficulty of envisioning a plate with very minimal carbohydrates, making this transition seem daunting. A prevailing emotional response during this phase is fear, which can manifest in various forms. Patients may experience fears related to judgment, standing out, missing out on experiences, being perceived as difficult, fearing failure or success, anticipating restrictions, and worrying about future health implications. These fears add to the emotional distress already inherent in big lifestyle changes.

### Mental health variations in transitioning

Challenges encountered during the dietary transition can vary based on specific mental health conditions. For example, individuals with depression may face struggles related to low self-efficacy and may benefit from additional emotional support. Those with health-related anxiety may need assistance in managing physical sensations without undue anxiety. Recognizing these variations is crucial for providing tailored support and interventions.

It’s important to recognize that some individuals may face the added challenge of transitioning to a ketogenic diet while dealing with ultra-processed food addiction (12). Addressing this addiction alongside dietary changes is crucial for a holistic approach to their well-being. For some, particularly those with a history of processed food addiction, additional psychosocial support may be essential to facilitate a successful transition to a ketogenic diet. Recognizing this need and providing access to the necessary support can be critical for their treatment compliance and overall well-being. Future research in this area could focus on regular screening using well-established assessment instruments like the Yale Food Addiction Scale (YFAS) (13) at the beginning of diet initiation to identify subsets requiring additional psychological intervention and potentially enhance treatment outcomes.

Regardless of clinical practice orientation, the goal is to facilitate a sustainable transition. Patients benefit from assistance in planning food procurement, meal preparation, and participation in family events, fostering gradual mastery. While the initial weeks may present challenges, with effective support, patients often find that continuing with KMT becomes more manageable, leading to ongoing benefits. Patient failure to follow electrolyte supplementation instructions can result in unpleasant electrolyte imbalances. Currently, there are guidelines for electrolyte supplementation in epilepsy populations that clinicians can use in current practice when using KMT as a treatment for mental illness (14). It is not uncommon for patients to report transient issues during the initiation phase (15) that can be clinically managed.

### Mental health fluctuations

While KMT shows promising potential as a mental health intervention, it’s important to recognize that the initial phase of the diet may not be a linear journey toward improvement for all individuals. For example, patients with bipolar disorder can sometimes experience a temporary worsening of symptoms before sustained symptom improvements occur.

During this initiation phase, patients may require additional support and contact from their KMT treatment team. Prescribers, in particular, may need to make themselves more available for consultations or symptom monitoring and may need to provide bridge medications and be open to appropriate deprescription (16). Mental health professionals can play a vital role in providing ongoing support and monitoring of symptoms, which can be used to help prescribers assess the patient’s needs.

As of now, further research is needed to fully elucidate the underlying mechanisms and specific patient profiles that may be more prone to these fluctuations. Nevertheless, the potential benefits of KMT, including symptom reduction and even remission of serious mental illnesses, often outweigh these temporary challenges for patients.

### Societal pressures and relationship dynamics

Individuals from diverse ethnic backgrounds, for whom family and holiday gatherings are centered around traditional foods, have highlighted the unique challenges they face in their social groups. Some have found that educating family members over an extended period and taking proactive steps, such as bringing their KMT-friendly dishes to events, can help ease the transition and maintain familial bonds (17, 18).

Different age groups and demographic backgrounds may experience these societal pressures differently. Teenagers and young adults, for example, may grapple with the desire to conform to peer dietary norms, experiencing feelings of exclusion when abstaining from certain foods. Those who frequently dine out may encounter difficulties finding restaurants with suitable menu options or may face predetermined meal choices that do not meet KMT requirements.

Patients often describe societal pressures as a notable challenge when initiating and maintaining Ketogenic Metabolic Therapy (KMT) for mental health. They recount instances where omitting carbohydrates from meals or declining carbohydrate-rich dishes leads to unexpected and sometimes distressing interactions with friends and family. These reactions can range from intrusive questioning to outright disapproval. Patients have reported clashes with prevailing notions of a well-balanced diet and warnings from misinformed individuals. Sometimes, the shift into carbohydrate restriction can strain existing friendships and family relationships. The dynamic can be similar clinically to someone with alcohol use disorder attempting abstinence and trying to find ways to connect with their social drinking circle. Patients often express uncertainty about navigating these changing dynamics.

Societal pressure and the desire to feel included in social situations can put patients at risk for symptom relapse. Within my clinical practice, this has definitely been a factor. This does not mean that the diet is “unsustainable” so much as some patients may need an extended period of check-in and support to help them both make the connection between their symptom relapse and their going off the diet and to allow them to reinitiate therapy in a supportive and non-judgmental environment. It has been my experience that often, these relapses happen after an extended period of wellness and an exit from regular psychotherapy because of symptom remission. At this point, it is unclear which patients need or would benefit from extended check-ins or support or if such extended support would lead to reduced relapse of symptoms in mental health populations using KMT.

However, while societal influences can initially pose challenges and require careful planning and the development of tools to facilitate healthy boundaries and relationship behaviors, the significant benefits that patients experience with KMT often outweigh these pressures. As patients witness improvements in their mental well-being, they frequently view societal expectations as a minor inconvenience compared to the reduction of symptoms they experience.

Overall, what I see in my practice with patients is empowerment occurs when they experience that they can take active control of their health through dietary choices. I cannot express how important this is for a population that has been told they have a chronic, debilitating lifelong illness for which, in the past, only symptom management was possible.

### Benefits observed in clinical practice

In the realm of mental health care, the implementation of Ketogenic Metabolic Therapy (KMT) has brought forth a spectrum of benefits for my patients that extend far beyond merely symptom management. As a clinician with hands-on experience using ketogenic diets as a treatment for mental illness, I’ve had the privilege of witnessing these transformations firsthand in populations in which I had begun not to expect significant improvement, let alone the levels of remission that occurred.

Many of my patients using this treatment report remarkable improvement in their cognitive function. Any clinician who works with mental health populations knows that patients complain of cognitive symptoms as much, if not sometimes more so, than the mood symptoms that brought them for treatment (19). Most patients I have worked with using this treatment describe heightened focus, better decision-making capabilities, and a noticeable reduction in the pervasive cognitive impairment they describe as brain fog. For these individuals, regaining their cognitive capacity played a large role in them being able to reclaim control over their lives.

Another clinical observation pertains to emotional stability. Patients who have undergone KMT often report a significant stabilization in their moods. They describe an experience in which they are able to take life as it comes and be much less overwhelmed. What my patients describe is a newfound emotional resiliency that some are rediscovering and others are experiencing for the first time in their lives. The benefit of having a predictable and balanced emotional state, along with the improvements in cognitive functioning, has had profound ripple effects on their daily lives and relationships.

Beyond addressing mental health symptoms, KMT has often led to reports by my patients of more holistic improvements in their well-being. They report increased energy levels, better sleep patterns, and restorative rest, which for many has not occurred for decades with pharmaceutical assistance. These additional holistic benefits highlight the interconnectedness of mental and physical health and the potential that ketogenic diets hold as a comprehensive therapeutic approach for the complex populations and presentations we encounter as mental health professionals.

### The future of competent treatment teams for KMT implementation

Controlled research settings by design offer exemplary support and consideration for participants needed to establish the efficacy and safety of this therapy. Private clinicians may struggle to help patients put together competent and experienced treatment teams for their patients. Ideally, this would consist of prescribers trained in ketogenic diet-informed medication adjustment, mental health practitioners who understand their role in using their counseling skills to support diet adherence, and access to ketogenic diet-trained nutritionists and dieticians who are comfortable working with individuals suffering from mental illness. Luckily, more and more professionally accredited training programs are being developed and offered for practitioners in each of these roles, and I am of the optimistic outlook that access to professionals trained in this therapy will increase. Even one professional appropriately trained in one of these roles and encouraged in collaborative practices may be able to improve outcomes for patients using KMT as a treatment for mental illness.

### The value in capturing qualitative data

In my experience, the changes I see in my patients benefiting from this treatment are just not adequately quantifiable. While we can celebrate large quantitative differences in mood symptom checklists and objective clinical assessments as they are published in the peer-reviewed literature, I would implore researchers to also capture qualitative data in various forms. The analysis of qualitative data can help the mental health field determine many important outcome measures in terms of implementing the diet with different populations and conditions. There is valuable insight into its successful or unsuccessful implementation with different ages, diagnoses, socioeconomic status, and a variety of other variables that clinical psychologists or other qualitative researchers could mine for valuable insights (20). As the field of clinical psychology will undoubtedly attempt to incorporate KMT into biopsychosocial models of practice, further research and tailored supports are expected to emerge, offering valuable insights for specific diagnostic populations.

Researchers who do not have access to professionals in fields that specialize in qualitative analysis have a special opportunity to work together to advance clinical practice. Psychiatrists and other mental health professionals will benefit from the practical treatment knowledge that will inevitably come from such analysis with better and more targeted interventions to show for the effort made. The collection of qualitative data could even be used to project long-term benefits in terms of costs associated with the healthcare system or even quality-of-life measures within families and communities that have vast implications (21, 22) and may be of generational significance. Why would I make such a strong statement about the benefits of measuring qualitative data alongside the hard biological markers and quantitative data already being collected by eminent researchers in this field? Because due to my training in clinical psychology and human development, I understand intimately that when you improve the mood, cognitive function, and emotional capacity of a parent (23), partner, sibling, or child, the ripple effect within the well-being of that system and the community around them is exponential (24) and possibly one of our greatest challenges to measure and celebrate.

### Concluding thoughts

There is great potential for a combined effort between researchers and clinicians to advance the field of mental health. The integration of KMT into existing mental health treatment systems of care represents a significant advancement in the field. Moving forward, it is crucial that we continue conducting rigorous research, valuing the nuanced insights from individual experiences, and fostering interdisciplinary collaborations. Together, we all have significant roles to play in increasing access to patients who are suffering from symptoms of mental illness. These patients should have the right to access this form of care from the very beginning of their difficulties. It is my perspective that this should not become a treatment of last resort, reserved for those who have met the criteria as being “treatment resistant,” nor should it be seen as merely an adjunctive treatment that “helps” the existing standard of care. In my clinical experience, KMT has been the intervention with the most profound and powerful treatment benefits for patients with mental illness. For that reason, our efforts in the field to study it, master it, and bring it to the masses as a standard of care become a moral and ethical imperative to a population we have inadvertently underserved for far too long (25).

## Data availability statement

The original contributions presented in the study are included in the article/supplementary material, further inquiries can be directed to the corresponding author.

## Ethics statement

Written informed consent was obtained from the individual(s) for the publication of any potentially identifiable images or data included in this article.

## Author contributions

NL: Writing – original draft.
